# Lithium-induced nephropathy; One medication with multiple side effects: a case report

**DOI:** 10.1186/s12882-022-02934-0

**Published:** 2022-09-09

**Authors:** Pingchuan Zhang, Hardik Gandhi, Nader Kassis

**Affiliations:** 1 Division of Anatomic Pathology, Mayo Clinic, 1st ST SW, Rochester, MN 55905, 200 Rochester, United States; 2 Sparrow Medical Group, Sparrow Hospital, 1200 E. Michigan ave, Michigan-48912, Lansing, United States

**Keywords:** Case report, Lithium nephropathy, Lithium side effects, Minimal change disease, Chronic tubulointerstitial nephropathy

## Abstract

**Background:**

Lithium carbonate is commonly used in the treatment of bipolar disorder. A spectrum of side effects is associated with lithium, including nephrogenic diabetes insipidus, renal tubular acidosis, chronic tubulointerstitial nephropathy, and minimal change disease. Although the former three adverse effects are well-known, minimal change disease is relatively rare.

**Case presentation:**

We herein report a case of lithium therapy-induced minimal change disease with concurrent chronic tubulointerstitial nephropathy. A 66-year old man with bipolar disorder treated by lithium for 20 years, presented to the hospital with anasarca and decreased urine output for 4 weeks. The medical history also included hyperlipidemia, hypertension, and benign prostatic hyperplasia. Further laboratory investigation revealed elevated serum lithium (2.17 mmol/L), potassium (6.0 mmol/L), and creatinine levels (2.92 mg/dL), nephrotic range proteinuria, and hypoalbuminemia. Lithium was discontinued and the patient was treated with intravenous fluids. He underwent a kidney biopsy, which showed findings consistent with minimal change disease with concurrent acute tubular injury and chronic tubulointerstitial nephropathy. The patient was subsequently treated with steroids in an outpatient setting. He did not respond to the treatment, and hemodialysis was started.

**Conclusion:**

Based on the previously reported cases and review of literature, occurrence of lithium-associated minimal change nephropathy is rare. Patients with lithium-associated minimal change disease and acute tubular injury usually respond to discontinuation of lithium therapy and/or steroid treatment. In this case, minimal change nephropathy was steroid-resistant and kidney function of the patient reported here did not recover after 6-month follow-up. We postulated the underlying cause to be minimal change disease with chronic tubulointerstitial nephropathy due to long-term lithium use. This case provides an example of a rare side effect of lithium-induced minimal change nephropathy with chronic tubulointerstitial nephropathy in addition to its well-known complication of interstitial nephritis or diabetes insipidus. In our opinion, these patients likely have much worse clinical outcome.

## Background

Lithium carbonate is most commonly used in the treatment of bipolar disorder. According to National Ambulatory Medical Care Survey (NAMCS), 17.6% of patients with bipolar disorder in the 2013- 2016 period were treated with lithium [1]. Among multiple organ systems affected by lithium toxicity, nephrogenic diabetes insipidus, renal tubular acidosis, and chronic tubulointerstitial nephropathy are the most common adverse effects of chronic lithium use in the kidney [2]. Conversely, nephrotic syndrome, defined as proteinuria > 3 g/day, edema, hypoalbuminemia and hyperlipidemia, is a rare and not well-known complication associated with lithium use. In 1973, Duflot and colleagues [3] were the first to identify the association between therapeutic levels of lithium and nephrotic syndrome. Since then, < 30 lithium-induced nephrotic syndrome cases have been reported in the literature [4]. Most of these cases were associated with therapeutic serum lithium levels and there was prompt reversal of nephrotic syndrome upon cessation of lithium therapy [4–7].

Based on kidney biopsies, 14 of these patients had final diagnosis of minimal change disease and 4 focal segmental glomerulosclerosis (FSGS). Most of the cases reported in the literature developed nephrotic syndrome during the first year of lithium therapy. Here we report a patient, who presented to the clinic with supratherapeutic serum lithium levels, acute on chronic kidney injury, and nephrotic syndrome after 20 years of lithium therapy. A kidney biopsy reveals minimal change disease, acute tubular injury, and evidence of chronic tubulointerstitial nephropathy. With 3-month follow-up, the patient’s kidney function has not recovered, and, in our opinion, the synchronous occurrence of lithium-associated minimal change disease and chronic tubulointerstitial nephropathy may imply a worse clinical outcome.

### Case presentation

A 66-year old man with medical history of bipolar disorder treated with lithium therapy for 20 years presented to primary care doctor’s office with significant edema and decreased urine output for 4 weeks. The past medical history was also significant for hyperlipidemia, essential hypertension, chronic kidney disease and benign prostatic hyperplasia. His daily home medications included lithium 600 mg/day, atorvastatin 20 mg/day, bupropion 100 mg/day, lisinopril 10 mg/day, calcium carbonate 600 mg/day, cholecalciferol 2000 units daily, and omeprazole 20 mg/day. Further laboratory work revealed supratherapeutic lithium level of 2.17 mmol/L, acute kidney injury with creatinine of 2.92 mg/dL (baseline of 1.5–1.6 mg/dL), albumin of 1.9 g/dL (baseline of 3.8 g/dL) hyperkalemia with potassium of 6.0 mmol/L, and 24-h urine protein 7,382 mg (no albuminuria reported previously). The patient was advised to go to the emergency department for further evaluation.

On admission, the patient’s blood pressure was 110/80 mmHg, heart rate 77 beats/min, and respiratory rate 17 breaths /min. Past medical history, chart review and evaluations did not reveal any history of reported HIV, tuberculosis exposure or malignancy. Chest X-ray performed did not show any evidence of acute or latent TB. Physical examination revealed bilateral upper and lower extremity edema, scrotal edema, slow response to questions, but otherwise normal findings. Urinalysis showed absence of white and red blood cells. Kidney ultrasound was unremarkable with no kidney calculi or hydronephrosis.

**Fig. 1:**
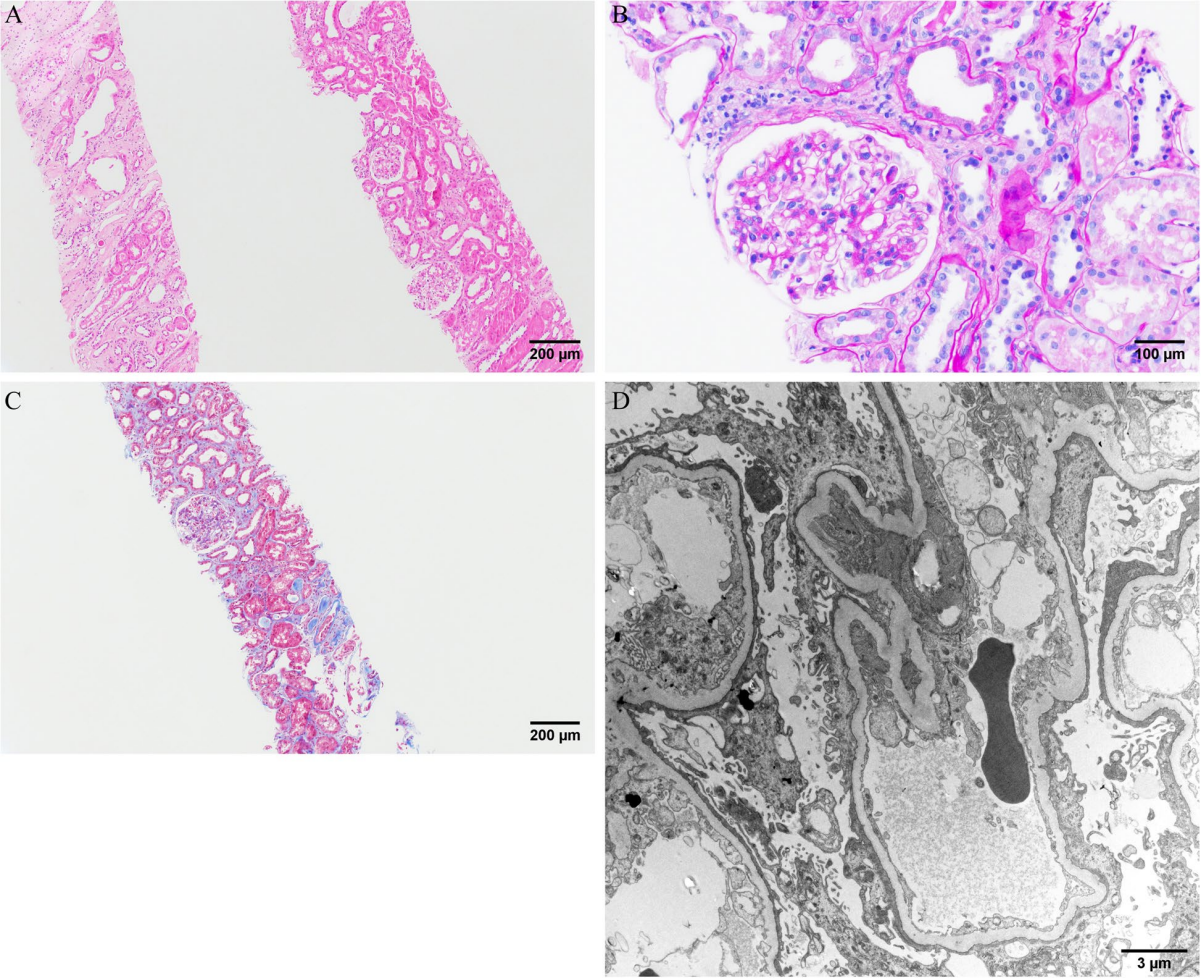
Light and electron microscopy Olympus BX53 and cellSense (Olympus Corporation, Center Valley, PA, USA) is used to capture the images. The images were obtained with eyepiece at 10X magnification and objective at 10X and 20X. The brightness and contrast were adjusted by using PowerPoint (Microsoft, Redmond, Washington, USA). No other downstream processing or averaging were applied to the images to enhance the resolution. **A **Haematoxylin and eosin (H&E) staining × 100: Two cores of kidney cortex and medulla show acute tubular injury and cystic dilatation of the distal tubules/ collecting ducts in the medulla. **B **Periodic Acid-Schiff (PAS) staining × 200: A glomerulus without segmental sclerosis or proliferative features. **C **Trichrome × 100: Mild tubular atrophy and interstitial fibrosis. **D **Electron microscopy × 7140: Diffuse podocyte foot process effacement with microvillous appearance.

Lithium therapy was discontinued immediately; the patient treated with intravenous fluids for hyperkalemia. Given the nephrotic range proteinuria and anasarca, he underwent a kidney biopsy. On light microscopy, approximately ten glomeruli were present for evaluation, five of which are globally sclerotic. The glomeruli showed no proliferative features or segmental sclerosis. Microscopic evaluation of tubules and interstitium revealed mild tubular atrophy and interstitial fibrosis. In the fibrotic areas, some of the glomeruli appeared ischemic. The glomeruli showed no FSGS or proliferative features. The immunofluorescence study was negative. Electron microscopy demonstrated severe diffuse and global foot process effacement. Taken together, the biopsy findings were consistent with a minimal change disease (Fig. 1).

The patient was discharged, and steroids were started outpatient based on the biopsy findings. His symptoms did not improve after discontinuing lithium therapy and steroid treatment; he continued to have significant edema. He presented to the hospital within 2 weeks with worsening kidney function, hyperkalemia, weight gain, edema in upper and lower extremities, and scrotal swelling. He had creatinine of 3.55 mg/dl, potassium of 5.1 mEq/dL and albumin of 1.6 g/dL. He continued to have nephrotic range proteinuria. He was treated with oral and intravenous diuretics with no relief in his anasarca. His steroids were also continued during the second admission. His creatinine continued to be elevated (2.94 mg/dl) and after discussing with the patient, a decision was made to initiate hemodialysis for anasarca refractory to diuretics and acute kidney injury. Patient continues to require hemodialysis 3 times a week and had progressed to end-stage renal disease (ESRD) with no improvement in kidney function as evidenced by significantly elevated intradialytic creatinine levels. The creatinine on 6-month follow-up was 5.86 mg/dl.

## Discussion and conclusion

Nephrogenic diabetes insipidus is the most common adverse effect associated with lithium therapy and may occur in up to 40% of the patients [2]; however, development of nephrotic syndrome is rare. There are only < 30 lithium-induced nephrotic syndrome cases reported in the literature, and most of these patients develop nephrotic syndrome in the first 1–2 years after initiation of lithium therapy [4–7]. Of note, glomerular filtration rate (GFR) in these patients usually does not fall below 40 ml/min/1.73m^2^. Among the cases reported, majority of the patients had complete reversal of proteinuria upon discontinuation of lithium therapy [4], although this is not the finding observed in the case reported here.

Although disputed in the past, the association between long term use of lithium and chronic kidney disease has been established by a French study [8]. In this study, Presne et. al. concluded that the progression rate of lithium-induced kidney dysfunction is related to the duration of lithium use, and chronic kidney disease is commonly seen in patients treated by lithium for 10–20 years. However, the molecular mechanisms are not well-established, and lithium-induced chronic tubulointerstitial nephropathy may be related to increased levels of inactive form of glycogen synthase kinase-3β (GSK- 3β) induced by elevated intracellular lithium concentration in distal tubules/collecting ducts [2]. Morphologically, Markowitz et. al [9]. observed that the most common kidney biopsy findings in the patients with lithium-associated kidney disease are tubular atrophy and interstitial fibrosis, cystic dilatation of distal tubules/collecting, and secondary segmental and global glomerulosclerosis due to chronic tubulointerstitial nephropathy. Furthermore, moderate elevation of the serum creatinine in 2.0 mg/dl range may represent irreversible progression of kidney injury even with prompt withdrawal of lithium therapy, and the initial serum creatinine at the time of biopsy was the only significant predictor of progression to end-stage renal disease (ESRD). The study indicated that only 1out of 10 patients progressed to ESRD with initial serum creatinine at the time of biopsy of < 2.5 mg/dl as compared to 7 of 9 patients progressed to ESRD with > 2.5 mg/dl. Focal segmental glomerulosclerosis is not a significant predictor of progression.

Subnephrotic range proteinuria, resulted from secondary glomerular damage, is a relatively common adverse effect of chronic lithium use, and it is estimated that up to 42% of patients on lithium may have > 1.0 g/day proteinuria. However, lithium-associated minimal change disease is rare, and the underlying mechanisms are far from clear. As lithium can modulate the phosphoinositol pathway, it is hypothesized that, through this pathway, lithium may activate T lymphocytes to release cytokines such as tumor necrosis factor, resulting in podocyte injury and minimal change disease [7].

Given the clinical presence of supratherapeutic lithium level and nephrotic syndrome, the acute tubular injury in the current case is likely secondary to nephrotic proteinuria. In the setting of nephrotic syndrome, the acute tubular injury may occur due to nephrosarca, which is defined as interstitial edema of kidney parenchyma. The interstitial edema is induced by hypoalbuminemia, and leads to physical obstruction of vasculature and tubules [10]. The novelty of the case reported here is that we see lithium associated minimal change nephropathy, which is a rare side effect of lithium besides its multiple other more common side effects such as diabetes insipidus and interstitial nephritis. As evidenced by the kidney biopsy; the glomeruli showed no proliferative features or segmental sclerosis. Microscopic evaluation of tubules and interstitium revealed mild tubular atrophy and interstitial fibrosis and was consistent with minimal change disease. Patient’s creatinine continued to worsen along with anasarca despite discontinuation of lithium therapy and steroids treatment. The patient progressed to ESRD requiring hemodialysis and continues to require hemodialysis 3 days a week 6-month after initial diagnosis. As mentioned previously, the risk of ESRD increases when the serum creatinine is > 2.5 mg/dl at the time of kidney biopsy, which was the case in this patient (2.7 mg/dl).

Lithium therapy has been an effective medication in treating bipolar disorder; unfortunately, with its narrow therapeutic range, it is associated with undesirable adverse effects. This case as presented above, although rare, strengthens the association of lithium therapy and nephrotic syndrome. It has been recommended that there is benefit of regular monitoring of patients’ lithium level to prevent chronic kidney disease and progression to ESRD as seen in this patient. Our case provides an example of a unique clinical scenario of lithium-induced minimal change nephropathy along with and chronic tubulointerstitial nephropathy. In our opinion, this combination suggests a worse clinical outcome. It is still needed to further confirm this impression from a larger clinical cohort.

## Data Availability

The data and patient medical information used during the current case report are available from the corresponding author upon reasonable request.

## Abbreviations

NAMCS: National ambulatory medical care survey
FSGS: Focal segmental glomerulosclerosis
ESRD: End-stage renal disease
GFR: Glomerular filtration rate
GSK- 3β: Glycogen synthase kinase-3β

## References

[1] Rhee T, Olfson M, Nierenberg  A, Wilkinson S (2020). 20-Year Trends in the Pharmacologic Treatment of Bipolar Disorder by Psychiatrists in Outpatient Care Settings. American Journal of Psychiatry.

[2] Grünfeld J, Rossier B (2009). Lithium nephrotoxicity revisited. Nature Reviews Nephrology.

[3] Duflot JP, Dore C, Fellion G, Kamba A, Morin F (1973). Use of lithium carbonate in a psychiatric institution. In Annales medico-psychologiques.

[4] Tandon P, Wong N, Zaltzman J (2015). Lithium-induced minimal change disease and acute kidney injury. North American Journal of Medical Sciences.

[5] Bosquet S, Descombes E, Gauthier T, Fellay G, Regamey C (1997). Nephrotic syndrome during lithium therapy. Nephrology Dialysis Transplantation.

[6] Sakarcan A, Thomas D, O’Reilly K, Richards R (2002). Lithium-induced nephrotic syndrome in a young pediatric patient. Pediatric Nephrology.

[7] Tam V, Green J, Schwieger J, Cohen A (1996). Nephrotic syndrome and renal insufficiency associated with lithium therapy. American Journal of Kidney Diseases.

[8] Presne C, Fakhouri F, Noël L, Stengel B, Even C, Kreis H (2003). Lithium-induced nephropathy: Rate of progression and prognostic factors. Kidney International.

[9] Markowitz GS, Radhakrishnan JA, Kambham N, Valeri AM, Hines WH, D'AGATI VD (2000). Lithium nephrotoxicity: a progressive combined glomerular and tubulointerstitial nephropathy. Journal of the American Society of Nephrology.

[10] Furuya R, Kumagai H, Ikegaya N, Kobayashi S, Kimura M, Hishida A (1993). Reversible Acute Renal Failure in Idiopathic Nephrotic Syndrome. Internal Medicine.

